# Significance of serum Myostatin in hemodialysis patients

**DOI:** 10.1186/s12882-019-1647-9

**Published:** 2019-12-11

**Authors:** Pasquale Esposito, Yuri Battaglia, Edoardo La Porta, Maria Antonietta Grignano, Elena Caramella, Alessando Avella, Sabrina Peressini, Nicodemo Sessa, Riccardo Albertini, Giuseppe Di Natali, Claudio Lisi, Marilena Gregorini, Teresa Rampino

**Affiliations:** 1 Unit of Nephrology, Dialysis and Transplantation, Fondazione IRCCS Policlinico San Matteo, and University of Pavia, Pavia, Italy; 2 Department of Internal Medicine, Division of Nephrology, Dialysis and Transplantation, University of Genoa and IRCCS Ospedale Policlinico San Martino, Genoa, Italy; 3 Department of Specialized Medicine, Division of Nephrology and Dialysis, Hospital-University St. Anna, Ferrara, Italy; 4 Clinical Chemistry Laboratory Fondazione IRCCS Policlinico San Matteo, Pavia, Italy; 5 Physical Medicine and Rehabilitation Unit, Fondazione IRCCS Policlinico S. Matteo, Pavia, Italy

**Keywords:** Hemodialysis, Myostatin, Malnutrition, Muscle wasting, Bioimpedance analysis

## Abstract

**Background:**

Malnutrition and muscle wasting are common in haemodialysis (HD) patients. Their pathogenesis is complex and involves many molecules including Myostatin (Mstn), which acts as a negative regulator of skeletal muscle. The characterisation of Mstn as a biomarker of malnutrition could be useful in the prevention and management of this condition. Previous studies have reported no conclusive results on the actual relationship between serum Mstn and wasting and malnutrition. So, in this study, we evaluated Mstn profile in a cohort of regular HD patients.

**Methods:**

We performed a cross-sectional study, enrolling 37 patients undergoing bicarbonate-HD (BHD) or haemodiafiltration (HDF) at least for six months. 20 sex-matched healthy subjects comprised the control group. Mstn serum levels were evaluated by ELISA before and after HD. We collected clinical and biochemical data, evaluated insulin resistance, body composition, malnutrition [by Malnutrition Inflammation Score (MIS)] and tested muscle function (by hand-grip strength, six-minute walking test and a questionnaire on fatigue).

**Results:**

Mstn levels were not significantly different between HD patients and controls (4.7 ± 2.8 vs 4.5 ± 1.3 ng/ml). In addition, while a decrease in Mstn was observed after HD treatment, there were no differences between BHD and HDF. In whole group of HD patients Mstn was positively correlated with muscle mass (r = 0.82, *p* < 0.001) and inversely correlated with age (r = − 0.63, *p* < 0.01) and MIS (r = − 0.39, *p* = 0.01). No correlations were found between Mstn and insulin resistance, such as between Mstn levels and parameters of muscle strength and fatigue. In multivariate analysis, Mstn resulted inversely correlated with fat body content (β = − 1.055, *p* = 0.002).

**Conclusions:**

Circulating Mstn is related to muscle mass and nutritional status in HD patients, suggesting that it may have a role in the regulation of skeletal muscle and metabolic processes. However, also considering the lack of difference of serum Mstn between healthy controls and HD patients and the absence of correlations with muscle function tests, our findings do not support the use of circulating Mstn as a biomarker of muscle wasting and malnutrition in HD.

## Background

Patients suffering from chronic kidney disease (CKD), mainly those undergoing hemodialysis (HD), often present malnutrition and muscle wasting, which directly correlate with morbidity and mortality [1]. Aetiology and pathophysiology of these conditions are complex and multifactorial, involving physical inactivity, insulin resistance, nutritional and hormonal changes and loss of muscle fibres [2]. Due to the relevant clinical impact of malnutrition and wasting, many attempts have been made to find a comprehensive definition of these conditions, in order to standardize diagnostic and therapeutic approaches. The different definitions took into consideration the levels of putative serum malnutrition markers (such as albumin and prealbumin), body mass index (BMI), the analysis of body composition and the evaluation of nutritional status by specific questionnaires (such as Subjective Global Assessment, SGA) [3]. However, while it is clear that an adequate evaluation of nutritional status requires a multistep approach, on the other hand the individuation of feasible and reproducible markers, allowing early diagnosis and monitoring of malnutrition and muscle wasting, is potentially useful [4].

Recently, the study of molecular and cellular mechanisms involved in the regulation of energy and muscular homeostasis has gained much interest. Myokines are molecules produced and released by skeletal muscle cells with systemic and paracrine actions, related to the activation of intracellular signalling pathways. These molecules, which include Myostatin, Irisin and IL-6, may have positive or negative effects on muscle growth and regulate relevant processes, such as increase fat oxidation, insulin sensitivity and inflammation [5].

The Growth Differentiation Factor (GDF)-8/Myostatin (Mstn) is a member of the Transforming Growth Factor (TGF)-β superfamily, primarily expressed in skeletal muscle cells and found also in other different cells and tissues, such as cardiomyocytes, macrophages and vessels. It is synthesized as a 376 amino acid pre-propeptide, then processed into an inhibitory propeptide of 242 amino acids and an active peptide of 110 amino acids [6].

Mstn exerts its effects in both autocrine and paracrine ways by binding a cell-bound Activin type II receptor 2B (ActRIIB) which, assembling itself with ALK4 or ALK5, leads to the activation of intracellular signalling pathways, including Smad 2/3 and Akt [7].

In skeletal muscle, Mstn limits muscle growth and promotes protein breakdown and its inhibitory effects have been described in both experimental models and clinical settings, with several studies demonstrating its increased expression in atrophic muscle and chronic diseases [8].

Although its action on skeletal muscle candidates serum Mstn as a potential biomarker for muscle wasting, the relationship between serum Mstn and skeletal muscle mass is still unclear.

Indeed, while the muscle-wasting effects of tissue myostatin are well established, many studies investigating serum Mstn in different disease conditions gave conflicting results, showing that Mstn may be both directly or inversely related to muscle mass or muscle wasting [9, 10].

In CKD patients an up-regulation of Mstn gene expression in skeletal muscle has been found, which was related to IL-6 expression, suggesting a link between Mstn and microinflammation [11]. Moreover, it has also been recently described that uremic toxins may accelerate muscle atrophy, by inducing Mstn expression [12]. However, only few studies have been focused on the evaluation of serum Mstn in CKD patients [13, 14].

Therefore, in this study we tried to characterize the profile of circulating Mstn and investigate its potentiality as a biomarker of malnutrition and muscle wasting in HD patients.

## Methods

### Study design

Adult (> 18 years) maintenance HD patients who had undergone HD for at least 6 months were enrolled in a cross-sectional. We excluded patients with: i) acute diseases, such as infections or immunological disorders, ii) immunosuppressive therapy, iii) history of transplantation or cancer*.* The control group was constituted by sex-matched healthy subjects.

We enrolled patients undergoing standard low-flux bicarbonate hemodialysis (BHD) or on-line hemodiafiltration (HDF) in a 2:1 ratio. BHD was performed with cellulose-based membranes using a blood flow rate of 300–350 mL/min (DICEA®,©Baxter International, IL, USA), while HDF was performed with high-flux membranes using a convective volume of 25–30% (FX100 High−Flux®,©Fresenius Medical Bad Hamburg, Germany).

For each patient we collected: i) clinical data, including age, dialysis modality, dialysis vintage and body mass index (BMI), and ii) biochemical data, such as pre-dialysis potassium, phosphate, transferrin, albumin, and C-reactive protein (CRP) serum levels. McAuley index (McA) = exp. [2.63–0.28 ln (insulin in mU/l) – 0.31ln (triglycerides in mmol/l)] was used to define insulin resistance (IR), considering a diagnostic cut-off point of ≤5.8 [15]. Serum Mstn level was tested by ELISA (Quantikine; R&D Systems, Minneapolis, MN, USA; detection limit 5.3 pg/ml), at the beginning and at the end of the hemodialysis session.

The study was performed according to the Declaration of Helsinki and was approved by the local Ethics Committee (protocol n. 9358/2015). All participants provided written informed consent before the enrollment.

### Body composition and nutritional evaluations

Body composition was studied by Body Composition Monitor (BCM, FMC, Bad Homburg, Germany). Measurements were taken before the HD treatment with the patient supine; electrodes were attached to the hand and foot on the same side of the body. As previously reported, a 3-compartment model of the body composition was applied. This model provides data on overhydration (OH), lean tissue index (LTI) and fat tissue index (FTI), normalized to height squared [16, 17].

Malnutrition-Inflammation Score (MIS) was used to assess nutritional status. It consists of ten items: modification in end-dialysis dry weight, dietary intake, comorbidities, functional capacity, gastrointestinal symptoms, BMI, loss of subcutaneous fat, decreased fat stores or/and signs of sarcopenia (according to SGA), serum albumin and total iron-binding capacity. Each item can present four levels of severity, from 0 (normal) to 3 (severely abnormal). Therefore, the MIS score can range from 0 to 30, with a higher score reflecting greater malnutrition and inflammation severity [18]. A score of 5 or above was considered to be indicative of malnutrition.

### Muscle function tests

All functional assessments were conducted by two trained assessors before the beginning of the HD session, in a quiet environment, using a standardized protocol, and included the dynamometer handgrip strength (HGS), the 6 min walking test (6MWT) and the Fatigue Severity Scale (FSS) [19]. The HGS was measured on the non-fistula arm using a Jamar hand dynamometer, considering the highest HGS value after three trials (with a one-minute pause between trials) [20].

6MWT was performed according to the American Thoracic Society guidelines. The respondents were asked to walk for 6 min along a 30 m corridor under medical supervision, at the normal pace they used daily. Test results consisted of the total covered distance, measured in m (with an accuracy of 1 m) [21].

Finally, muscular fatigue was assessed by FSS, a 9-item self-report questionnaire where each item is scored 1–7. The total score range from 9 to 63 and a score > 36 was considered pathological.

### Statistical analysis

Data are presented as mean ± standard deviation (SD) or interquartile ranges (IQR), if not normally distributed (as evaluated by Shapiro Test).

Analysis of variance (ANOVA), Student t-test or nonparametric Mann-Whitney test, were used to assess the differences among control group and HD patients. Spearman-Rho was used to assess the correlations between Mstn and clinical and laboratory variables, while logistic regression models were used to analyze the associations (Stata 13.1, Stata Corporation, College Station, Texas, United States). A 2-tailed *P* value < 0.05 was considered statistically significant.

## Results

### Patient characteristics

We enrolled 37 HD patients (69.6 ± 15 years, 14 females) with a dialysis vintage of 35 (19.5–48) months. At the time of enrollment, 24 patients (65%) were undergoing thrice-weekly 4-h BHD, while 13 patients (35%) HDF.

32 patients (86%) were hypertensive, 12 patients (32%) were diabetic and 12 patients (32%) were active smokers. The main underlying nephropathies included glomerulonephritis, nephroangiosclerosis, diabetic nephropathy and adult dominant polycystic kidney disease.

Mean BMI was 26.4 ± 4.2 kg/m^2^, while mean pre-dialytic potassium, albumin and CRP were 4.6 ± 0.7 mEq/l, 32 ± 4 (3.2 ± 0.4) g/dl and 0.76 (0.25–1.2) mg/dl, respectively. Mean McAuley index was 5.6 ± 2. Considering a cut-off of ≤5.8, 23 patients (62%) resulted insulin-resistant.

20 healthy subjects (48.5 ± 10 years, *p* < 0.01 vs HD, 8 females), with normal renal function (creatinine 78.2 ± 16 μmol/L) and mean BMI of 27 ± 2 kg/m2, constituted the control I group.

Whole patient characteristics are shown in Table 1.

**Table 1:** Patient characteristics at the time of clinical observation

	Total population
N	37
Gender (M/F)	23/14
Age, (years)	69.6 ± 15
BMI, (kg/m^2^)	26.4 ± 4.2
Diabetes, n (%)	12 (32)
Time on dialysis, months (IQR)	35 (19.5–48)
Type of dialysis, n (%)	
BHD, n (%)	24 (65)
HDF, n (%)	13 (35)
Serum potassium (mEq/L)	4.6 ± 0.7
Serum albumin (g/L)	32 ± 0.4
Prealbumin (g/L)	0.3 ± 0.08
Total cholesterol (mmol/L)	3.6 ± 0.9
Triglycerides (mmol/L)	1.8 ± 0.8
Phosphate (mmol/L)	1.6 ± 0.5
McAuley index	5.6 ± 2
CRP (mg/dl), IQR	0.76 (0.25–1.2)
Transferrin (g/L)	1.7 ± 0.3
spKT/V	1.66 ± 0.3

All values were determined in predialysis

Abbreviations: Body mass index (BMI), Bicarbonate-hemodialysis (BHD); Hemodiafiltration (HDF), C-reactive protein (CRP), standard pool KT/V (spKT/V)

### Myostatin profile

**Fig. 1:**
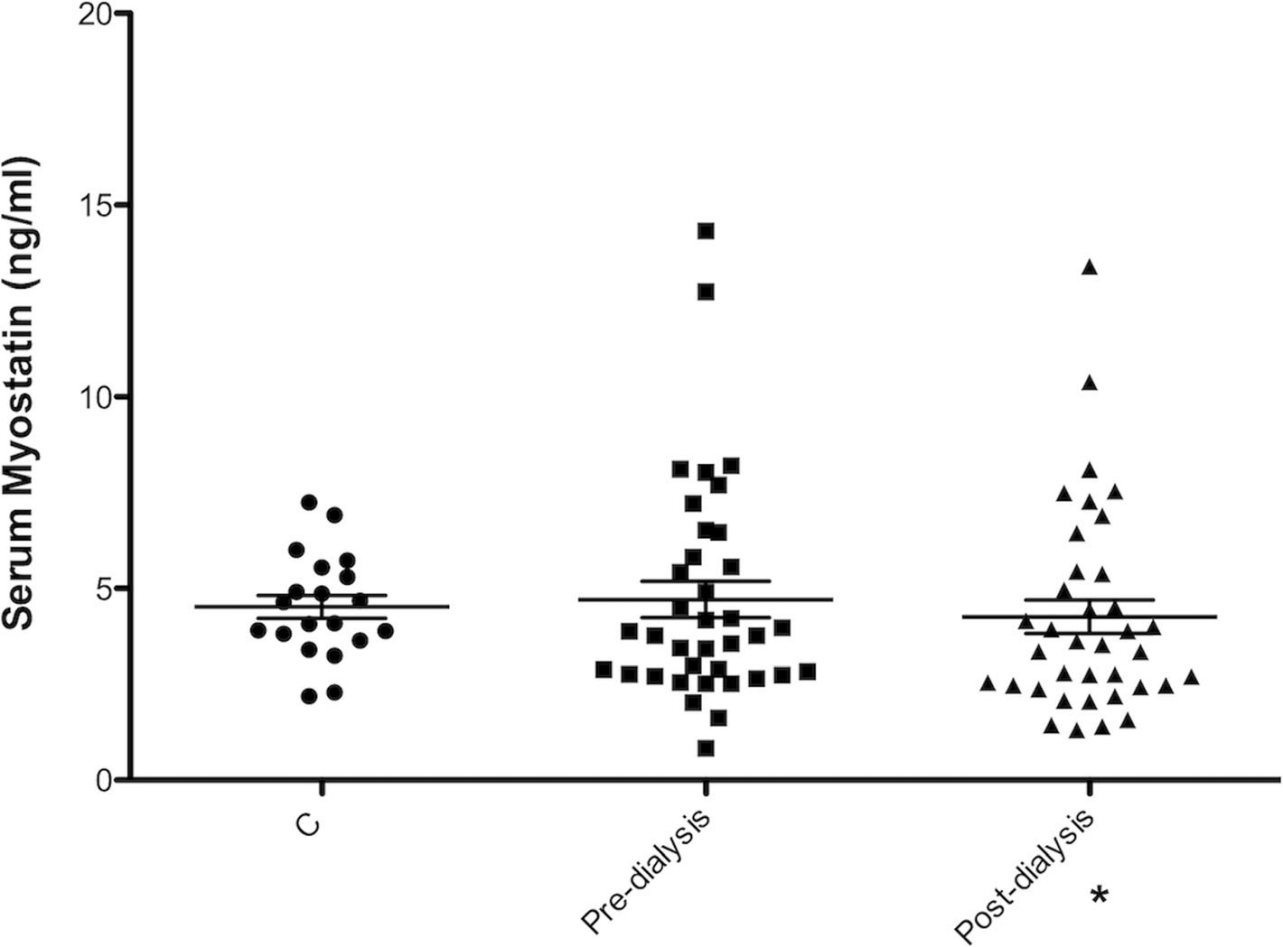
Serum Myostatin levels in HD patients. There were not significant differences in serum myostatin levels between healthy control subjects (C) and patients undergoing hemodialysis. Post-dialysis there was a significant decrease in Myostatin levels compared with pre-dialysis values. * *p* < 0.05

There was no significant difference in serum Mstn levels between pre-dialysis HD and Control groups (4.7 ± 2.8 vs 4.4 ± 1.3 ng/ml, *p* = 0.8). Moreover, in the HD group post-dialysis Mstn levels resulted significantly lower than pre-dialysis ones (4.2 ± 2.6 ng/ml, *p* = 0.02). Finally, taking into consideration the different dialytic modalities, we found no differences in Mstn levels, comparing patients undergoing BHD or HDF (4.7 ± 2.8 vs 4.6 ± 3 ng/ml) (Fig. 1).

### Nutritional parameters and functional tests

As recorded by BCM, mean LTI and FTI were 12.3 ± 2.9 and 14 ± 5.9 kg/m2, respectively. There were no significant differences between female and male patients.

Mean MIS score was 14 ± 3. Considering a MIS cut-off ≥5, we demonstrated the presence of malnutrition in all the evaluated HD patients.

All participants completed the functional assessment. Mean HGS was 25.1 ± 9 kg, but it was significantly lower in women than in men (19.8 ± 5.9 vs 27.7 ± 9.3 kg, *p* < 0.05). The average distance achieved in the 6MWT was 365 ± 79 m.

Finally, mean FSS was 45 ± 11, presenting a clear pathological value (i.e. > 36) in 31 patients (83%) (Table 2).

**Table 2:** Body composition and muscle function tests at the time of clinical observation

	Total population
N	37
Serum Myostatin (ng/ml)	4.7 ± 2.8
LTI, (kg/m^2^)	12.3 ± 2.9
FTI, (kg/m^2^)	14 ± 5.9
OH (L), IQRs	0.5 (−0.9_1.2)
MIS	14 ± 3
HGS (kg)	25.1 ± 9
Male (n.24)	27.7 ± 9.3
Female (n.13)	19.8 ± 5.9*
6MWT (m)	365 ± 79
FSS	45 ± 11

All values were determined in predialysis. * p < 0.05 vs males

Abbreviations: Lean Tissue Index (LTI), Fat tissue index (FTI), Body mass index (BMI), Overhydration (OH), Malnutrition Inflammation Score (MIS), Hand-grip strength (HGS), 6-min walking test (6MWT), Fatigue Severity Scale (FSS)

### Correlations

**Fig. 2:**
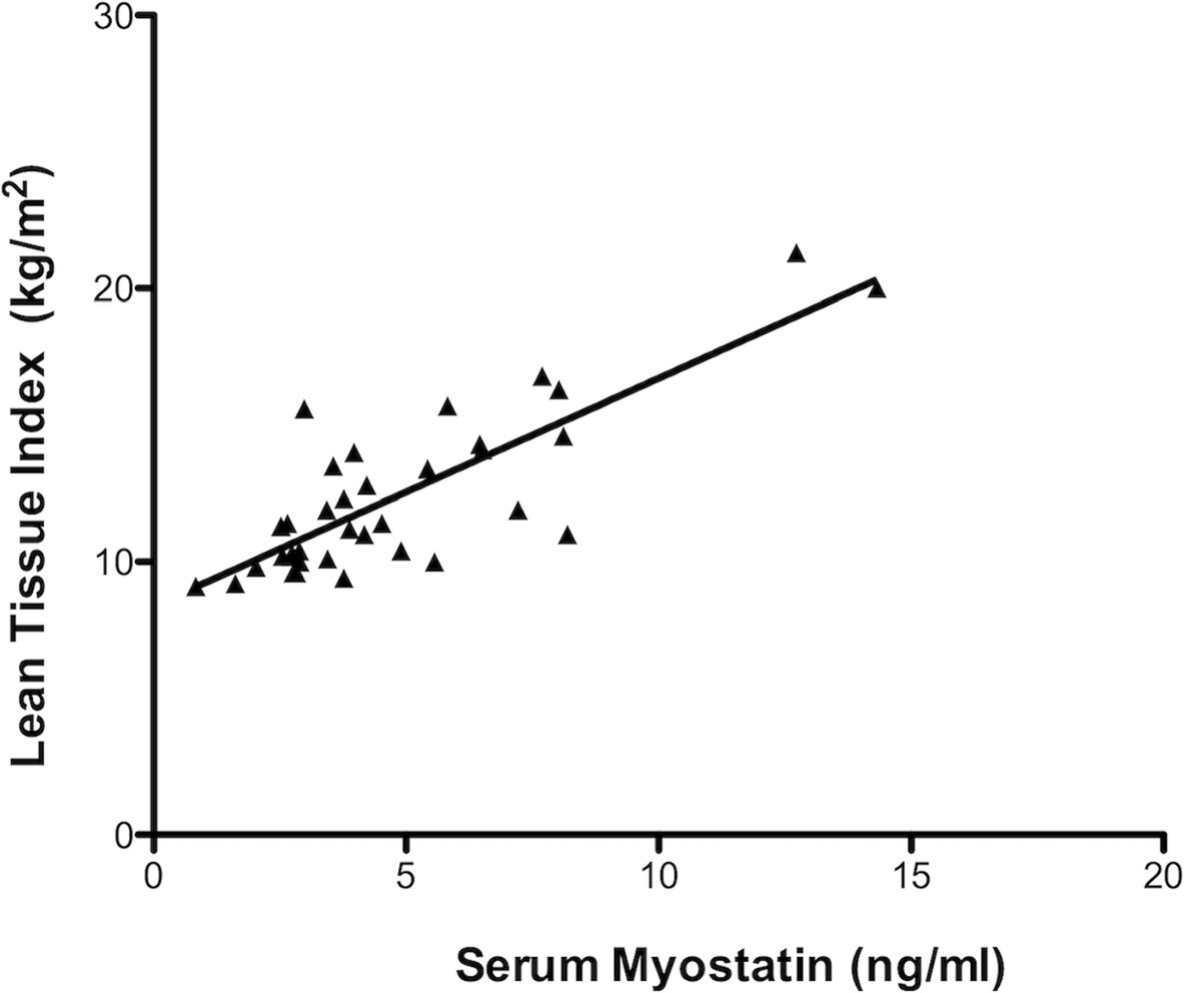
Correlation between serum Myostatin and muscle mass in HD patients. There was a direct correlation between serum Mstn and muscle mass (expressed as Lean Tissue Index-LTI). Linear regression analysis, r = 0.67, *p* < 0.0001

Correlation analysis showed a direct association between pre-dialysis Mstn and LTI, albumin and phosphate serum levels (Fig. 2), whereas there was a significant inverse association with age, BMI, FTI and MIS. Moreover, MIS resulted inversely correlated with serum albumin levels and LTI, while was directly correlated with age, CRP levels, OH state (r = 0.44) and fatigue, expressed as FSS (r = 0.37, *p* = 0.02) (Table 3). McAuley index, defining insulin resistance (i.e. lower values correspond to increased risk of IR), was inversely correlated with BMI and FTI and directly related to HD vintage and OH. HGS was directly correlated with muscle mass, expressed as LTI (r = 0.41, *p* = 0.01), and inversely correlated with age (r = − 0.35, *p* = 0.04).

**Table 3:** Correlations among Myostatin serum levels, nutritional and functional parameters in HD patients

*N* = 37	Age	HD-age	MSTN	Phosphate	BMI	LTI	FTI	CRP	Albumin	McA	MIS
Age	1										
HD-age	0.12	1									
MSTN	−0.63§	0.01	1								
Phosphate	− 0.44+	− 0.08	0.4*	1							
BMI	0.06	−0.09	− 0.4*	− 0.06	1						
LTI	−0.68 §	0.143	0.82 §	0.38*	−0.45	1					
FTI	0.28	−0.2	−0.65 §	− 0.18	− 0.18§	−0.7 §	1				
CRP	0.27	0.17	−0.19	−0.12	0.05	−0.17	−0.06	1			
Albumin	−0.09	−0.04	0.35*	0.02	0.14	0.3	−0.01	−0.07	1		
McA	0.19	0.43+	0.06	−0.09	−0.54+	0.18	−0.45+	− 0.006	−0.18	1	
MIS	0.47+	−0.06	−0.39*	− 0.04	−0.29	− 0.44+	−0.02	0.37*	−0.43*	0.12	1

Correlation coefficients are shown. * p < 0.05; + p < 0.01; § *p* < 0.001

Abbreviations: Hemodialysis (HD), Myostatin (Mstn), Body mass index (BMI), Fat tissue index (FTI), lean tissue index (LTI), Overhydration (OH), C-reactive protein (CRP), McAuley index (McA), Malnutrition Inflammation Score (MIS)

Finally, in multivariate analysis, after adjustment for age, sex and HD vintage, Mstn resulted inversely associated with FTI (β = − 1.055, *p* = 0.002).

## Discussion

In this study, we tried to define the meaning of serum Mstn in HD patients.

First, we evaluated the circulating levels of Mstn and the potential effect of dialytic treatment on these levels, taking into consideration different dialysis techniques (BHD vs HDF). We found no differences in Mstn levels between HD patients and healthy control, while there was a significant slight decrease of Mstn after a single HD session, confirming our previous data of a potential modulation of serum Mstn by HD treatment [22]. However, in contrast with other reports, we did not observe significant differences in Mstn levels between BHD and HDF [14, 22].

This finding could be explained by many factors, including the diverse assays used to measure circulating Mstn, the different study design (crossover vs observational) and the small number of patients enrolled in these studies that may have not allowed to discriminate little differences between the two HD modalities.

Furthermore, basing on the known inhibitory effects of Mstn on skeletal muscle growth, we investigated the hypothetical use of serum Mstn level as a biomarker for muscle wasting, a very common condition in HD patients, significantly related to relevant clinical consequences.

Therefore, we studied circulating Mstn in relation to nutritional and metabolic parameters and muscle function tests. Interestingly, we found that in HD patients serum Mstn resulted directly correlated with muscle mass, evaluated by BIA. Coherently, circulating Mstn resulted inversely correlated with BMI, fat body content and age, indicating that obese and older patients with low muscle mass present low circulating Mstn levels. This data was also reinforced by the evidence of direct correlation of Mstn with albumin and phosphate levels, often used as markers of good nutritional status, and its inverse correlation with malnutrition, evaluated by MIS.

All these findings are in apparent contradiction with those reported in skeletal muscle and suggest that circulating Mstn reflects muscle mass content rather than muscle wasting. On the other hand, this data is not so surprising, since the actual relationship between serum Mstn and skeletal muscle mass in humans remains controversial. Indeed, while there is evidence of an inverse correlation between serum Mstn and skeletal muscle mass in elderly and patients affected by chronic diseases, it has been also reported that low serum Mstn levels are associated with low skeletal muscle mass in in heart failure patients with cachexia, such as in healthy old adults [9, 10, 23, 24]. Similarly, in HD patients, while Mstn has been found to be overexpressed in muscle and strictly linked to inflammation and muscle atrophy, the analysis of serum Mstn levels has shown contrasting results [13, 14, 22, 25]. Therefore, it seems there could be a discrepancy between muscular and circulating Mstn, whose explanation is not so clear. First, it is possible that circulating Mstn not necessarily reflects its intramuscular concentration, since the protein may be also produced from other tissues or undergoes degradation. Secondly, it is conceivable that other extracellular matrix proteins might interact with Mstn and Mstn-linked molecules, regulating Mstn expression and TGF-β signalling pathway in muscle cells [26].

However, when we looked at the functional meaning of serum Mstn, we did not find correlations among serum Mstn and functional tests exploring muscle strength, endurance and fatigue.

Analogously, when we studied the metabolic parameters, we did not find significant correlations among circulating Mstn and metabolic pathways, including lipids and insulin resistance (IR), evaluated by McAuley index. Also in this case, as well as for that reported for muscle mass, the relationship between circulating Mstn and IR is controversial and matter of debate. Indeed, while previous studies showed elevated Mstn levels in obese patients with hyperinsulinemia and IR, configuring a condition in which IR might potentiate the inhibitory effect of Mstn on muscle growth [27], high serum Mstn has also been reported to be associated with favourable metabolic profiles and a lower prevalence of metabolic syndrome [28].

Therefore, overall these findings indicate that while circulating Mstn seems to reflect the muscle mass, its actual clinical significance and utility in HD patients remains questionable.

Beyond data on Mstn, our study highlighted some other collateral findings that are worthy to be considered. First of all, we confirmed that malnutrition is very common in HD patients. In our study this condition, which actually implies a complex and multifaceted pathogenesis, resulted related to age, inflammation (evaluated as CRP levels) and overhydration (OH) state. In particular, the correlation of OH with malnutrition and inflammation has been also reported in other studies [29] and is of peculiar interest in HD patients, since volume control is one of the main problems in the daily management of these patients.

Regarding muscle function tests, we found that HGS was directly correlated with muscle mass, as also previously reported, and inversely related to age, while self-reported muscle fatigue correlated with malnutrition, which may probably represent the functional correspondent.

We are aware that our study presents some limitations, mainly due to the observational design and the small number of patients evaluated with control subjects younger than HD patients.

Indeed, while a single determination of circulating Mstn is related to muscle mass, it is possible that the periodical monitoring (i.e. the time trend) of Mstn levels could provide useful information about muscle loss and progression of cachexia over the time.

Moreover, it is conceivable that the study of complex processes, such as muscle wasting and malnutrition in HD, should not be limited to the evaluation of a specific marker.

For example, beyond Mstn, other molecules, such as cytokines, activins and follistatin, may regulate muscle growth and metabolism [30]. In particular, among them, Activin A, which shares the receptor with Mstn (i.e. ActRIIB) and has been related with muscle loss in cancer, seems worthy of being investigated as an additional biomarker of muscle wasting, also in HD patients [31, 32].

Finally, it should be underlined that the study of circulating Mstn could also be made difficult by technical limitations, since in the Literature there is some concern about the use of ELISA-based approaches to measure Mstn concentration, mainly because Mstn immunoreactivity does not necessarily equal to its bioactivity [33].

## Conclusions

In conclusion, we think that the relationship between Mstn and muscle mass and nutritional status candidate circulating Mstn as an interesting new player in the regulation of skeletal muscle trophism. This is particularly relevant, since Mstn is currently object of many researches on the potential role of its pharmacological inhibition, aiming to promote muscle mass increase and improve the metabolic profile and frailty in different disease conditions [34–36]. However, current evidence is not strong enough to support the use of serum Mstn to diagnose muscle wasting and malnutrition or to monitor the responses to the treatments in HD patients. Therefore, further studies, possibly prospective and performed with more accurate analytical methods (like mass spectrometry), are needed to elucidate the potentialities of circulating Mstn as a biomarker and its utility in detecting patients at risk for wasting.

## Data Availability

The datasets generated and/or analysed during the current study are available from the corresponding author on reasonable request.

## Abbreviations

6MWT: 6-min walking test
BHD: Bicarbonate hemodialysis
*BMI*: Body mass index
CKD: Chronic kidney disease
CRP: C-reactive protein
ELISA: Enzyme-linked immunosorbent assay
*ESRD*: End-stage renal disease
FSS: Fatigue Severity Scale
FTI: Fat tissue index
HD: Hemodialysis
HGS: Hand-grip strength
LTI: Lean Tissue Index
McA: McAuley index
MIS: Malnutrition Inflammation Score
Mstn: Myostatin
OH: Overhydration
spKT/V: standard pool KT/V
